# A novel germline *PALB2 *deletion in Polish breast and ovarian cancer patients

**DOI:** 10.1186/1471-2350-11-20

**Published:** 2010-02-02

**Authors:** Agnieszka Dansonka-Mieszkowska, Anna Kluska, Joanna Moes, Michalina Dabrowska, Dorota Nowakowska, Anna Niwinska, Pawel Derlatka, Krzysztof Cendrowski, Jolanta Kupryjanczyk

**Affiliations:** 1 Department of Molecular Pathology, the Maria Sklodowska-Curie Memorial Cancer Center and Institute of Oncology, Roentgena 5, 02-781 Warsaw, Poland; 2 Department of Endocrinology, the Maria Sklodowska-Curie Memorial Cancer Center and Institute of Oncology, Roentgena 5, 02-781 Warsaw, Poland; 3 Department of Genetic Counselling, the Maria Sklodowska-Curie Memorial Cancer Center and Institute of Oncology, Roentgena 5, 02-781 Warsaw, Poland; 4 Department of Breast Tumors and Reconstructive Surgery, the Maria Sklodowska-Curie Memorial Cancer Center and Institute of Oncology, Roentgena 5, 02-781 Warsaw, Poland; 5 Department of Gynecologic Oncology, the Maria Sklodowska-Curie Memorial Cancer Center and Institute of Oncology, Roentgena 5, 02-781 Warsaw, Poland; 6 Chair and Department of Obstetrics, Gynecology and Oncology, IInd Faculty of Medicine, Warsaw Medical University and Brodnowski Hospital, Warsaw, Poland

## Abstract

**Background:**

PALB2 protein was recently identified as a partner of BRCA1 and BRCA2 which determines their proper function in DNA repair.

**Methods:**

Initially, the entire coding sequence of the *PALB2 *gene with exon/intron boundaries was evaluated by the PCR-SSCP and direct sequencing methods on 70 ovarian carcinomas. Sequence variants of interest were further studied on enlarged groups of ovarian carcinomas (total 339 non-consecutive ovarian carcinomas), blood samples from 334 consecutive sporadic and 648 consecutive familial breast cancer patients, and 1310 healthy controls from central Poland.

**Results:**

Ten types of sequence variants were detected, and among them four novel polymorphisms: c.2996+58T>C in intron 9; c.505C>A (p.L169I), c.618T>G (p.L206L), both in exon 4; and c.2135C>T (A712V) in exon 5 of the *PALB2 *gene. Another two polymorphisms, c.212-58A>C and c.2014G>C (E672Q) were always detected together, both in cancer (7.5% of patients) and control samples (4.9% of controls, p = 0.2). A novel germline truncating mutation, c.509_510delGA (p.R170fs) was found in exon 4: in 2 of 339 (0.6%) unrelated ovarian cancer patients, in 4 of 648 (0.6%) unrelated familial breast cancer patients, and in 1 of 1310 controls (0.08%, p = 0.1, p = 0.044, respectively). One ovarian cancer patient with the *PALB2 *mutation had also a germline nonsense mutation of the *BRCA2 *gene.

**Conclusions:**

The c.509_510delGA is a novel *PALB2 *mutation that increases the risk of familial breast cancer. Occurrence of the same *PALB2 *alteration in seven unrelated women suggests that c.509_510delGA (p.R170fs) is a recurrent mutation for Polish population.

## Background

PALB2 protein [OMIM #610355, a partner and localizer of BRCA2] was recently identified in a complex with BRCA2 (Breast Cancer 2) protein [1]. PALB2 supports BRCA2 stability and determines its localization in the nucleus after DNA damage [1]. Relocation of PALB2 and BRCA2 to damaged chromatin is regulated by BRCA1 (Breast Cancer 1) protein [2]. These three proteins form a complex in which PALB2 acts as a bridge between BRCA1 and BRCA2 [2]. In cells depleted of PALB2, DNA repair pathway dependent on the BRCA1/2 is disrupted [1,2]. This suggests that inactivation of the *PALB2 *gene may cause similar biological and phenotypic changes as inactivation of the *BRCA1 *or *BRCA2 *genes; the latter is responsible for a fraction of breast and ovarian cancers, and a dysfunction of BRCA2 alone - for the Fanconi anemia (FA) syndrome.

FA syndrome is a genetic disorder caused by germline biallelic mutations in one of 13 genes (including *FANCD1*/*BRCA2 *and *FANCN*/*PALB2*) of the so-called FA/BRCA pathway; this pathway controls the repair of double strand-breaks and the response to DNA crosslinking agents [3,4]. Studies on FA patients provide evidence that biallelic mutations of the *PALB2 *gene are responsible for one of the syndrome's subtypes, which is very similar to the one caused by inactivation of the *BRCA2 *gene [5,6].

Mutations of the *BRCA1 *and *BRCA2 *genes are the best known alterations of the FA/BRCA pathway. Heterozygous germline mutations of these genes predispose to breast and ovarian cancers. The cumulative risk of ovarian cancer to age 70 years for women with a *BRCA1 *or *BRCA2 *mutation is estimated at about 40% and 10%, respectively [7]. The cooperation between BRCA2 and PALB2 during the DNA repair process and similarity in clinical features of the FA syndrome caused by biallelic mutations of *BRCA2 *and *PALB2 *genes suggest the role of *PALB2 *alterations in carcinogenesis. To date, *PALB2 *alterations have been associated with familial breast [8-13] and pancreatic [14,15] cancers. To our knowledge, *PALB2 *gene has not been extensively studied in ovarian cancer patients.

We demonstrate a novel inherited monoallelic deletion of the *PALB2 *gene in ovarian and breast cancer patients which seems to be a recurrent mutation for the population of central Poland.

## Methods

### Subjects

The study was performed on a series of 339 non-consecutive patients with ovarian cancer and 982 patients with breast cancer; all patients were Caucasian women from central Poland.

### Ovarian cancer patients

The analysis was performed on tumor fragments (n = 339); patients' blood samples were used to confirm germline origin of the detected variants only. The samples were collected in two Warsaw hospitals (Institute of Oncology and Brodnowski Hospital) between the years 1995 and 2007. These hospitals treat cancer patients from central Poland. Mean age of patients was 56 years (range 17-88). Distribution of cases in appropriate age intervals in the group studied was comparable to that in the whole population of ovarian cancer patients in Poland [16]. Tumors were classified histologically according to the criteria of the World Health Organisation [17]. There were 231 (68%) serous carcinomas, 33 (10%) endometrioid, 15 (4%) mucinous, 26 (8%) clear cell, 17 (5%) undifferentiated and 17 (5%) other type carcinomas. *BRCA1 *mutational status (exons 2, 5, 11 and 20) had been previously determined in the majority of ovarian carcinomas (n = 243), and 39 (16%) were positive [18]. A few carcinomas were also studied for *BRCA2 *mutations in exons 2, 3, 11 (c.3035-6629) and 25 (Moes et al., data not published).

### Breast cancer patients

DNA from the peripheral blood leukocytes was analyzed in 334 sporadic and 648 familial breast cancer cases, the latter with at least one first-, second- or third-degree relative affected by breast and/or ovarian cancer. In 40 families with third-degree relative affected, additional criteria were employed: carcinomas developing in a proband and/or a relative before the age of 50, metachronous or synchronous cancers diagnosed in a proband or a relative, or non-breast and non-ovarian cancers diagnosed in first- or second-degree relatives. The probands were patients of the following departments of the Institute of Oncology: Breast Tumors and Reconstructive Surgery, and Genetic Counselling. *BRCA1 *mutations (exons 2, 5, 11 and 20) had been previously detected for the purpose of counselling in 75 of 982 breast cancer patients (8.3%). The mean age of patients was 50 (range 21-81) and 48 years (range 24-85) for the sporadic and familial breast cancers, respectively.

### Controls

As a control, we used 1310 blood samples from unrelated Caucasian women from central Poland. These samples were anonymously collected in Warsaw blood donation centers between November 2005 and February 2009 (n = 1142). A part of control blood samples (n = 168) were collected among employees and cancer-free patients from Brodnowski Hospital in Warsaw. Mean age of females from the control group was 32 years (range 18 to 75).

Patients gave their written informed consent to be included in the study. Anonymous blood donors gave informed consent. The study was approved by the bioethics committee of the Institute of Oncology (ref. no. 39/2007).

### Molecular genetic methods

All 13 *PALB2 *exons with intron/exon boundaries were initially screened for variants in 70 non-consecutive ovarian carcinomas by the PCR-SSCP and sequencing methods. Sequence variants of interest were further studied in larger groups of ovarian carcinomas and in control blood samples (see: Results). The germline origin of changes detected in ovarian carcinomas was confirmed in blood samples from those patients. The analyses were performed in the Department of Molecular Pathology. Blood from breast cancer patients was screened only for the c.509_510delGA *PALB2 *deletion in the Department of Endocrinology, with the use of the dHPLC method.

### DNA extraction

Fresh ovarian cancer specimens were snap-frozen and stored at -68°C. Cryostat sections were cut and evaluated by a pathologist (JK) as to the sufficient content of tumor tissue. Blood samples were collected in vials which contained EDTA, frozen and stored at -68°C. DNA from both ovarian carcinomas and blood samples was extracted with the use of proteinase K and the QIAamp Mini kit (Qiagen), and stored in the AE buffer (Qiagen). DNA from blood samples of breast cancer patients was isolated with the Genomic Midi AX kit (A & A Biotechnology, Gdansk, Poland).

### PCR - Polymerase Chain Reaction

DNA fragments were amplified by the PCR method. Primers (presented in Table 1) were designed using the free Primer3 software [19] and the *PALB2 *genomic sequence, obtained from the NCBI genome browser [GenBank: NG_007406.1].

**Table 1:** *PALB2 *gene primers sequences

Exon	Forward primer	Reverse primer
1	GATTTAATTGGCCGGAGTTT	GGGTGGTCAGATGATACTGC

2 - 3	CTTGCCCAGTATTGTTTGGTG	GCAGGCATAAGTGAATGGTC

4a	TCATCTGCCTGAATGAAATG	TGAGTGAATCAGTGCCAAAG

4b	CAAGAACATTTTCCCCACAG	GGAGGAATGTGTTCAAGGTG

4c	AGGGCGACTACAGTTCCTTT	TGCAGAAAGAGGAGAGGTTG

4d	TTGATGGCAGGAATGAAAAT	GCAACTGCCTTCCTAGACAA

4e	ATGCACAGGACAACCAAGTT	TTGGCCCTGTCACTTTTTAG

5a	TGTCTGTTTTGTTGGGTTTTG	TCCATGCGTTTAGGACTCAG

5b	TGCTCAGAAAAACCAGTGGA	AGCAAGTTCGTCCAGCAAC

5c	CCCTCAAGGCTCCTATGAAA	GGCATTTCATTCCTTCAGAGA

6	GTGGGTAATGCAGGCAGAC	TGTTTTTCTGAATCTGTTTACCAA

7	TTTGCATAAAACAGCACTCG	TTTGGTAAGCTGCCCATCTA

8	TGATAAATTTTGGAAAATCTGGA	CTGCACTTAAAACCAGCTGAC

9	ACTCCTCACATCACCCCATT	TATTACACCCCCAGCACAGA

10	CGGAGAAGGGCTACCTAGAG	GCAACACAAAACCACAATCA

11	TTGTTTGTTGGAAGAATGTGA	CGGGGAAGGTTTGTTCATTA

12	AGAGCCTATCGGTCATTGCT	TTCAGAATGTCCCACCCATA

13	GGATTTTTGTTCCTGTTGCTG	TCTCCTTTATATTTAAAACTCCAAAAA

PCR mixture was prepared according to a standard method (Applied Biosystems PCR kit). PCR reaction was carried out for 36 cycles in a programmable thermal cycler (Biometra) with denaturation at 94°C, annealing at 56-58°C (depending on the exon) and extension at 72°C, for 30 sec each.

### Single strand conformational polymorphism analysis (SSCP)

All thirteen coding exons of the *PALB2 *gene were screened by the SSCP method. In our experience, this method detects 90% of all alterations, and 100% of deletions and insertions [20]. PCR products were denatured with 0.1 M NaOH containing 2 mM EDTA, at 55°C for 15 min. Immediately after 95% formamide, 0.05% xylene cyanol and 0.05% bromphenol blue were added, the samples were loaded to polyacrylamide gels (1:39 *N, N'*-methylenebisacrylamide to acrylamide in 0.5 × TBE with 10% glycerol). Electrophoresis was performed at 100 V, for 16-24 hours at room temperature. DNA bands were visualised by the silver-staining method compilated from several procedures.

### Denaturing High-Performance Liquid Chromatography

DNA from breast cancer patients was screened for *PALB2 *c.509_510delGA only (with Ex 4b-F and Ex 4b-R primers), using the dHPLC method carried out on automated dHPLC instrumentation (Transgenomic Inc). Crude PCR products were eluted with linear acetonitryle gradient. The gradient and the temperature required for successful resolution of heteroduplex molecules was determined with the use of the dHPLC melting algorithm (Transgenomic Inc). Mutation positive and negative controls for the screened mutation were always run with each set of samples. The heterozygous profiles were identified by visual inspection of the chromatograms, on the basis of appearance of additional, earlier eluting peaks.

### Sequencing

We sequenced all variants detected by the SSCP and dHPLC. PCR products were purified by exonuclease I and alkaline phosphatase treatment, and then sequenced with the use of fluorescent automated method (BigDye Terminator Cycle Sequencing Kit v3.1, Applied Biosystems) with ABI PRISM 377 DNA sequencer.

### PALB2 Haplotyping

DNA extracted from blood samples of *PALB2 *mutation carriers was genotyped with the use of three markers applied by Foulkes et al. [8]: D16S841 [UniSTS:2638], D16S403 [UniSTS:150021], and D16S417 [UniSTS:67206]. Primer sequences were obtained from the NCBI [21] UniSTS database. Marker and *PALB2 *gene positions were reckoned from NCBI [21] Homo sapiens chromosome 16 genomic contig (reference sequence: NT_010393.16). The loci were amplified by PCR (as described earlier, at the annealing temperature of 58°C) with the use of fluorescently labelled (6-FAM dye) forward primer. PCR products were diluted in sterile water, in a volume depending on reaction efficiency. The dilution at the volume of 0.8 μl was mixed with 8 μl of formamide (SIGMA) and 0.4 μl of the standard (Gene Scan 500 ROX, Applied Biosystems). The mixture was denaturated for 5 min at 95°C and then cooled on ice. Electrophoresis was performed in the ABI PRISM 377 DNA sequencer. Data were analyzed using the Peak Scanner Software v1.0 (Applied Biosystems).

### Immunohistochemical stainings

Markers of basal/luminal types were evaluated immunohistochemically with the use of the following antibodies: anti-CK5/6 (DAKO, clone D5/16B4), anti-CK14 (Novocastra, clone NCL-L-LL002), anti-CK17 (DAKO, clone IR620), anti-EGFR (DAKO, clone K1994), anti-CD117 (DAKO, clone A4502). All immunostainings were performed against negative controls. Non-neoplastic mammary gland structures served as intrinsic positive controls.

### Statistical analysis

Differences between the compared groups were analyzed by the two-sided Fisher's exact test [22]. The level of statistical significance was set at <0.05.

## Results

The initial screening of 70 ovarian carcinomas revealed nine substitutions and one deletion of the *PALB2 *gene. These alterations, and the final number of the samples studied, are shown in Table 2.

**Table 2:** Sequence variants in the *PALB2 *gene (in brackets - groups on which they were studied)

Exon	Nucleotide change	Effect	Frequency in cancers	Frequency in healthy controls	SNP Id# or references
4*	**c.509-510delGA**	**p.R170fs**	ovarian cancer **0.6% (2/339)** familial breast cancer **0.6% (4/648)** sporadic breast cancer **0 (0/334)**	**0.08% (1/1310)**	**novel**

Analyzed in ovarian cancers only					

4*	c.505C>A	p.L169I	0	0.08% (1/1310)	novel

4*	c.618T>G	p.L206L	0	0.08% (1/1310)	novel

4*	c.656A>G	p.D219G	0	0.08% (1/1310)	rs45594034:A>G

5**	c.2014G>C	p.E672Q	7.5% (15/200)	4.9% (16/326)	rs45532440:G>C

5	c.2135C>T	p.A712V	0.5% (1/200)	0.3% (1/326)	novel

12	c.3300T>G	p.T1100T	4.3% (3/70)	Not studied	rs45516100:T>G

Intron					

3**	c.212-58A>C		7.5% (15/200)	4.9% (16/326)	[10,12]

6	c.2586+58C>T		5.7% (4/70)	Not studied	rs249954:C>T

9	c.2996+58T>C		1.4% (1/70)	Not studied	novel

(GenBank accession number: genomic DNA- NG_007406.1, mRNA and protein- NM_024675.3)

^# ^NCBI's [21] SNP database

* confirmed by the SSCP and sequencing in separate PCR reactions

** in our study these polymorphisms were always detected together

### Substitutions (studied in ovarian cancer patients only)

Nine substitutions were detected, four of which are novel (Table 2). Two sequence variants, i.e. c.212-58A>C in intron 3 and c.2014G>C in exon 5 were always detected together, both in cancer and control samples (p = 0.2). The majority of carcinomas and controls carrying these alterations were heterozygous for exon 5 and intron 3. Only one tumor and one control DNA were homozygous, and there were alleles C in both polymorphic sites.

### c.509_510delGA deletion

**Figure 1:**
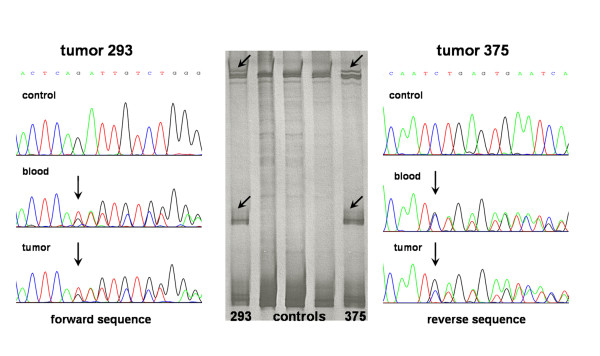
**The c.509_510delGA detected in the *PALB2 *gene in ovarian carcinomas**. SSCP gel (in the middle) - shifted bands are indicated by arrows; on sequencing diagrams the deletion site is indicated by arrows.

We discovered a novel 2 base-pair deletion c.509_510delGA in exon 4 of the *PALB2 *gene (Figure 1, Table 2). It resulted in p.R170fs and created a premature stop codon at position 183.

### Ovarian cancer patients

The alteration c.509_510delGA was detected in 2 of 339 ovarian cancers (0.6%) and in 1 of 1310 controls (0.08%; p = 0.1). The deletion was present in both tumor and blood DNA of the two patients. The analysis of sequencing diagrams (Figure 1) of tumor sample number 375 (about 30% of stromal cell contamination) and blood DNA of the same patient suggests the loss of heterozygosity at this locus. LOH was also observed at two of the three studied microsatellite loci (D16S481 and D16S403, data not shown).

One of the two patients with the deletion (tumor number 293) was diagnosed with a poorly differentiated endometrioid carcinoma at the age of 61 (The Federation Internationale de Gynecologie et d'Obstetrique staging system - FIGO IIIC, histological grade 3). She reported a history of cancers in her father's family. The father had a stomach cancer diagnosed at the age of 67, his sister had a lung cancer (age unknown), his brother had an unknown cancer at an unknown age. This patient also carried a nonsense germline mutation i.e. c.C4513T (Q1429X) in the *BRCA2 *gene.

The other patient (tumor number 375) was diagnosed with a poorly differentiated serous carcinoma at the age of 54 (FIGO IIIC, histological grade 3). She reported a history of cancers in her mother and her mother's sisters (thyroid and pancreatic carcinomas mentioned, age unknown). No mutations in exons 2, 3, 11 (c.3035-6629) and 25 of the *BRCA2 *gene were found.

Both of these patients died and we did not have a chance to confirm these data.

### Breast cancer patients

Screening of 982 consecutive sporadic or familial breast cancer patients for c.509_510delGA resulted in detection of the additional four mutation carriers. The deletion was found in 4/648 (0.6%) familial breast cancer patients versus 1/1310 controls (p = 0.044).

Histopathological features of breast carcinomas from four of the deletion carriers are presented in Table 3. All but one had triple (ER, PR, HER2)-negative phenotype. To characterize breast carcinomas with the *PALB2 *mutation more specifically, we evaluated the expression of CK5/6, CK14, CK17, EGFR and CD117 (Table 3). Two carcinomas were of the basal type and two of the luminal type.

**Table 3:** Characteristics of breast cancers with c.509_510delGA deletion in the *PALB2 *gene

**Proband no**.	Age	Type, grade	ER, PR	HER2	CK5/6, CK17, EGFR	CK14	CD117
**802**	53	ductal, G2	**-**	**-**	+	+	**-**

**2076**	62	ductal, G3	**-**	**-**	**-**	+	**-**

**893**	44	ductal, G3 bifocal	+	-	-	-	+

**1540**	47	medullary	-	-	+	+	- (focal +)

ER-estrogen receptor, PR- progesterone receptor, HER2-human epidermal growth factor receptor 2, CK- cytokeratin, EGFR- epidermal growth factor receptor, CD117-C-kit receptor

**Figure 2:**
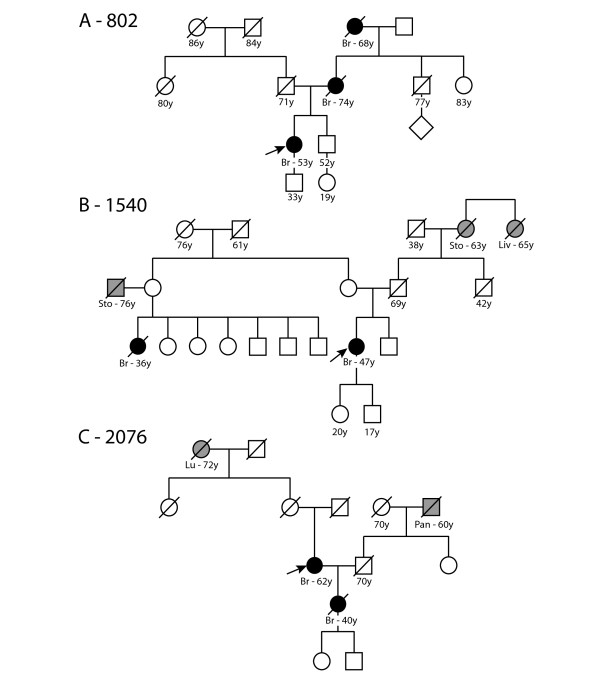
**Pedigrees of breast cancer families with *PALB2 *mutations**. Probands with confirmed mutation are indicated by an arrow. Br -breast cancer, Sto -stomach cancer, Liv -liver cancer, Lu - lung cancer, Pan - pancreatic cancer.

Pedigree diagrams were available for three patients (Figure 2). The pedigree was not available for the patient number 893, but she stated a breast cancer in her grandmother at the age of 87.

### Haplotype analysis

Genotyping of the seven c.509_510delGA deletion carriers was performed with three microsatellite markers: D16S417 which is distal to *PALB2*, and D16S481 and D16S403 that are proximal to this gene. Genotypes are presented in Table 4. There was no common haplotype for all *PALB2 *mutations carriers with regard to the three markers analyzed.

**Table 4:** Results of genotyping of *PALB2 *mutation carriers

**Proband no**.	Marker position bp	D16S403 22,977,651	D16S481 23,188,290	*PALB2* 23,554,483*	D16S417 23,717,034
**293**	Ov. Ca.	2, 3	1, 2	c.509-510delGA	2, 4
**375**	Ov. Ca.	2, 6	1, 2	c.509-510delGA	2, 4
**ZB649**	Control	4, 3	1, 2	c.509-510delGA	3, 4
**802**	Br. Ca.	1, 6	3, 2	c.509-510delGA	2, 4
**2076**	Br. Ca.	4, 5	1, 1	c.509-510delGA	2, 1
**893**	Br. Ca.	4, 3	1, 2	c.509-510delGA	3, 4
**1540**	Br. Ca.	4, 3	1, 1	c.509-510delGA	2, 4

Alleles for each microsatellite marker were numbered according to the size. Ov. Ca. - ovarian cancer patients; Control - a *PALB2 *mutation carrier from the control group; Br. Ca. - breast cancer patients

* the position of *PALB2 *gene on chromosome 16 (NCBI reference sequence: NT_010393.16); the position of the deletion is 23,587,357 bp

## Discussion

We discovered a novel *PALB2 *germline deletion, c.509_510delGA, located in exon 4. It may be expected that due to this deletion PALB2 protein is shortened to 169 amino acids from 1186 amino acids of the wild type and it is devoid of C-terminal domain (with WD40 repeats). WD40 motifs are thought to be responsible for protein-protein interactions and they seem to be necessary for BRCA2/PALB2 complex formation [11]. It was shown that *PALB2 *mutants (229delT, 2521delA and c.1592delT) that lacked C-terminal domain had highly reduced BRCA2 binding capacity and were defective in the repair of double-strand breaks and mitomycin C-induced damages [10,11]. To date, all *PALB2 *gene alterations detected in families with breast cancer or FA disease were frameshift or nonsense changes, leading to the expression of a truncated protein. These data strongly support the assumption that the deletion detected in this study results in the production of an inactive protein. Silent and missense sequence variants of the *PALB2 *gene have been detected in previous studies in both cancer samples and in control tissues, but none of those variants have been strongly associated with cancer susceptibility [9,10,12].

Our study proves that *PALB2 *alterations contribute to the familial but not to the sporadic breast cancer in Poland. This is in agreement with previous reports, in which alterations of the *PALB2 *gene were predominantly associated with familial breast cancer [9-13]. Some authors reported *PALB2 *mutations also in pancreatic cancer [14,15], male breast cancer [9] and in a single prostate cancer family [10]. Pancreatic cancer was mentioned at least twice in the family histories of our *PALB2 *mutation carriers.

The low frequency of *PALB2 *mutations (0.6%) observed in our study is similar to that noticed for breast cancer by other authors [8,9,11-13]. Only Erkko et al. [10] found a higher rate of mutations in the familial breast cancer: 2.7%. In their study, however, the rate of the mutations in the control population was also relatively high (0.2%). No other study except Erkko's et al. [10] and our study found *PALB2 *truncating mutations in control populations.

We are the first to present a *PALB2 *truncating mutation in a patient with medullary breast carcinoma. To date, such alterations have been detected predominantly in ductal breast carcinomas. In agreement with data presented by Heikkinen et al. [23], our study suggests that breast cancers with *PALB2 *mutations are predominantly triple-negative ones; however, their phenotype does not completely overlap with the basal type.

In our study, *PALB*2 alterations did not associate with ovarian cancer risk. In addition, the *PALB2 *deletion was accompanied by a germline *BRCA2 *nonsense mutation in one ovarian cancer patient (Moes et al., unpublished data). A similar observation was published by Heikkinen et al. [23] who found two breast cancer patients with a *PALB2 *deletion among 104 *BRCA2 *mutation carriers. Interestingly, the presence of these two alterations in carcinomas appears to be more frequent than just one occurring by chance, considering the low frequency of *PALB2 *and *BRCA2 *mutations in control populations.

To date, *PALB2 *gene alterations have not been extensively studied in ovarian cancer patients. Erkko et al. [24] found three (0.5%) ovarian cancer patients with *PALB2 *gene mutations in 593 unselected ovarian cancer cases screened for the *PALB2 *c.1592delT alteration, identified earlier in breast cancers [10]. Another dysfunction of *PALB2 *detected in ovarian cancer was its expression silencing by promoter hypermethylation (in 4 of 53 sporadic cases) [25]. These studies, together with our data suggest that PALB2 protein may be defective or insufficient in some rare ovarian carcinomas; however, a possible contribution of this insufficiency to ovarian cancer development needs elucidation.

It is unclear how the PALB2 deficiency contributes to cancer development. It has been suggested that PALB2 participates in breast carcinogenesis through haploinsufficiency and/or the dominant-negative effect. In the majority of breast cancers with *PALB2 *gene mutations, the loss of heterozygosity (LOH) was not observed [10,11]. The only evidence of deletion of the wild-type allele was presented by Garcia et al. [12]. In our study, there was a suspicion of LOH at *PALB2 *locus in one ovarian carcinoma with *PALB2 *deletion. The regular allele of *PALB2 *gene may also be eliminated by promoter hypermethylation (as above). Such dysfunction was revealed not only in ovarian but also in sporadic breast cancers [25]. Since wild-type PALB2 proteins oligomerize at damaged DNA [26], truncated PALB2 mutants may possibly complex with wild type protein, thus, disturbing its proper function. In addition, other molecular changes may cooperate with PALB2 deficiency in cancer development.

Some of *PALB2 *gene alterations in breast cancer were suggested to be founder mutations for other ethnic groups [8,10]. The presence of the same deletion in seven unrelated women in our study might suggest that this was a founder mutation for the examined population from central Poland. Although the genotype analysis of the mutation carriers showed differences in the haplotypes, one cannot exclude an ancient founder mutation. More detailed analysis is necessary to determine the origin of this alteration.

Our study had some limitations. The entire coding sequence of the *PALB2 *gene was screened in 70 non-consecutive ovarian cancers only; the sensitivity of the SSCP ranges from 70% to 95%, according to different publications [27,28], and it is 90% in our laboratory [20]. Thus, some *PALB2 *alterations (particularly of the missense type) could have been missed. Nevertheless, a practical value of this study is that the c.509_510delGA should be searched for in Polish families with breast cancer.

## Conclusions

The c.509_510delGA is a novel *PALB2 *mutation that increases the risk of familial breast cancer. Occurrence of the same *PALB2 *alteration in seven unrelated women suggests that c.509_510delGA (p.R170fs) is a recurrent mutation for population from Poland. It may be useful to include this *PALB2 *mutation to a list of alterations that should be searched for in Polish families with breast cancer.

## Competing interests

The authors declare that they have no competing interests.

## Authors' contributions

ADM designed and coordinated the study, carried out molecular analyses of ovarian cancers and controls, coordinated the collection of control blood samples, drafted the manuscript. AK carried out molecular analyses of breast cancer. JM participated in the design of the study, carried out a part of molecular analyses of ovarian cancers and controls. MD participated in molecular analyses of breast cancer. DN collected and characterized the breast cancer material, performed pedigree diagrams. AN collected and characterized the breast cancer material. PD participated in collection of ovarian cancer samples from the Institute of Oncology. KC participated in collection of ovarian cancer samples and controls from the Brodnowski Hospital. JK characterized the ovarian cancer material, analyzed immunohistochemical stainings, participated in the design and coordination of the study, critically reviewed and drafted the manuscript. All authors read and approved the final manuscript.

## Pre-publication history

The pre-publication history for this paper can be accessed here:

http://www.biomedcentral.com/1471-2350/11/20/prepub
